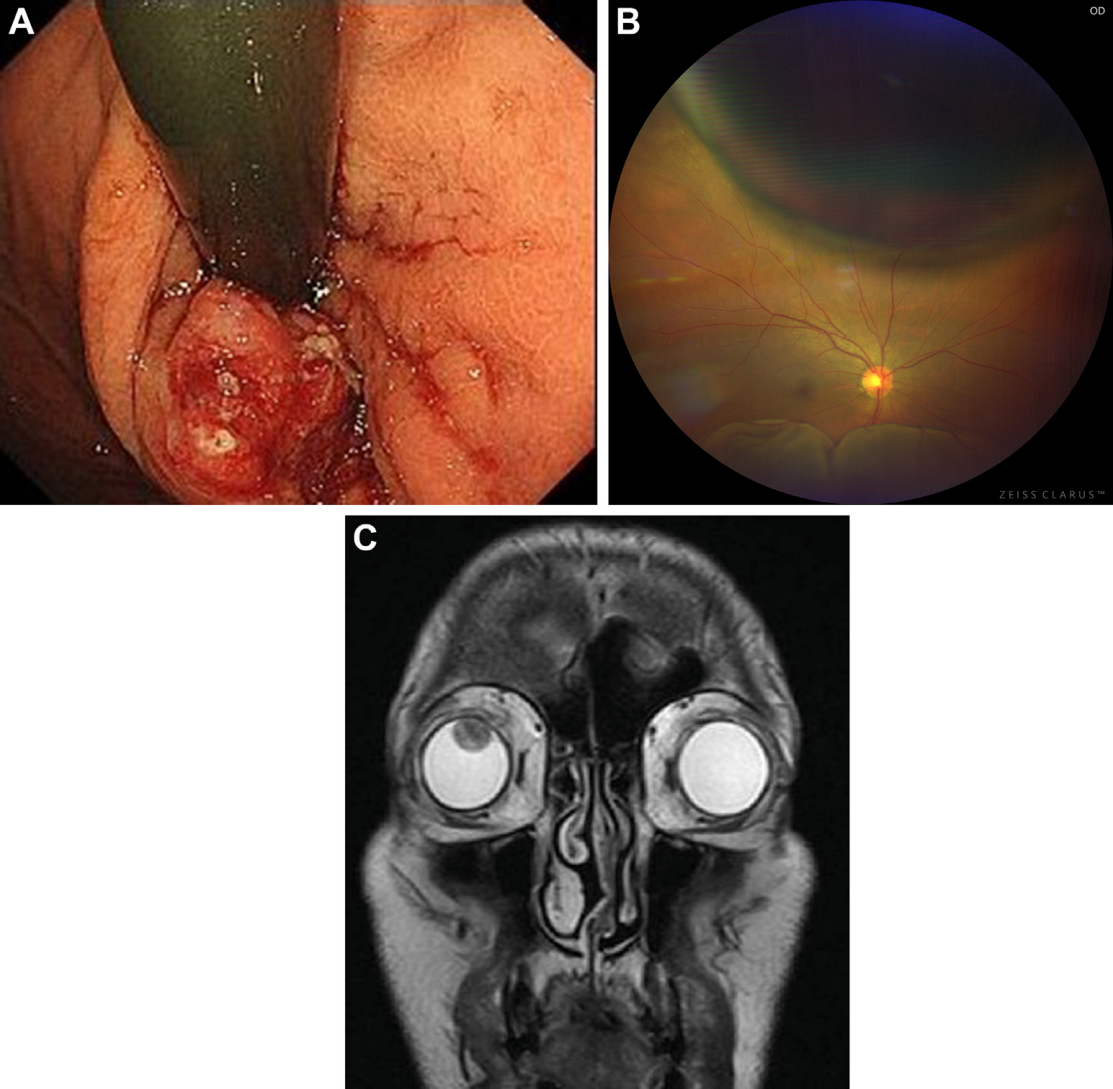# Choroid Metastasis From Human Epidermal Growth Factor Receptor Type 2-Positive Gastric Cancer

**DOI:** 10.1016/j.gastha.2022.09.002

**Published:** 2022-09-10

**Authors:** Tomonobu Koizumi, Noriko Yoshida, Kai Mizuhata

**Affiliations:** 1 Department of Hematology and Medical Oncology, Shinshu University School of Medicine, Matsumoto, Japan; 2 Department of Ophthalmology, Shinshu University School of Medicine, Matsumoto, Japan; 3 Department of Radiology, Shinshu University School of Medicine, Matsumoto, Japan

A 39-year-old man with human epidermal growth factor receptor type 2-positive gastric cancer with hepatic metastasis had been treated with first-line chemotherapy consisting of trastuzumab + S-1 + oxaliplatin followed by trastuzumab deruxtecan for 18 months. Endoscopic examination showed a mass in the esophagogastric junction (Figure A), but disease remained stable during chemotherapy. He was referred to the ophthalmology department of our hospital due to visual impairment in the right eye. The right eye showed a mass on the superior subretinal space at 1- to 3-o’clock position with secondary serous retinal detachment in the inferior eye (Figure B) suggesting a metastatic tumor. Coronal magnetic resonance imaging showed a smooth and sharply marginated tumor in the right eye with low-intensity signals on Dixon T2-weighted imaging (Figure C). Based on the clinical course, a diagnosis of choroid metastasis of gastric cancer was made. The tumor size in the right eye was reduced after radiotherapy (3 Gy × 10 fractions), but this failed to improve the visual impairment. Choroid metastasis in patients with gastric cancer is extremely rare. In addition, there have been no previous case reports regarding the development of choroid and/or eye metastasis in cases of human epidermal growth factor receptor type 2-positive gastric cancer.

**Figure:**